# Assessment of Sex, Age, and Metabolism Relationships to Serum Thyroid Concentrations in Retired Alaskan Husky Sled Dogs

**DOI:** 10.3389/fvets.2022.859066

**Published:** 2022-06-14

**Authors:** Margret Lenfest, John P. Loftus, Heather J. Huson, Andrei Gudkov, Katerina Andrianova, Daria Fleyshman, Joseph Wakshlag

**Affiliations:** ^1^Department of Clinical Sciences, Cornell University College of Veterinary Medicine, Ithaca, NY, United States; ^2^Department of Animal Sciences, Cornell University College of Agriculture and Life Sciences, Ithaca, NY, United States; ^3^Vaika Foundation, Buffalo, NY, United States

**Keywords:** Alaskan Husky, sled dog, metabolic energy, thyroid, free T4, total T4

## Abstract

Sled dogs are purpose-bred dogs selected for endurance work. Prior studies in racing dogs showed that serum thyroid parameters (total T4, free T4, and T3) are lower than the reference range in approximately 25% of dogs. Whether this is related to training, breeding, or body condition remains unclear. We hypothesized that retired sled dogs of normal body condition (9–13 years old) would have predominantly normal serum thyroid parameters and that serum thyroid status would be correlated to energy consumption based on metabolic body weight. Eighty-six sled dogs who were deemed healthy on physical exam, not on confounding medications, and without a prior diagnosis of hypothyroidism were included. All dogs' mean body condition scores were 5.1 ± 0.4 and body weight 24.5 ± 4.2 kg at fasting blood collection with stable dietary intake for 3 months before sampling. The total T4, free T4, and T3 serum concentrations were 23.4 ± 9.1 nmol/L, 9.53 ± 4.3 pmol/L, and 0.93 ± 0.39 nmol/L, respectively, with 38% lower than the reference range for total T4, 45% for free T4, and 37% for T3. All dogs were negative for thyroglobulin antibody, and TSH results were within normal ranges. Pearson's correlates based on kilocalories consumed on a metabolic body weight basis for total T4 (*R* = 0.14), free T4 (*R* = 0.01) and T3 (*R* = 0.23) showed poor correlation. No differences were observed between thyroid hormones and age, breed, or sex. Inactive, retired sled dogs can be misdiagnosed with hypothyroidism; therefore, our data suggests that misdiagnosis of hypothyroidism can occur and that the racing Alaskan sled dog has a unique reference range that should be considered when assessing serum thyroid status.

## Introduction

Thyroid hormones play several essential roles in the dog. Thyroid hormones have been proposed to increase most tissues' metabolic rate and oxygen consumption, induce positive inotropic and chronotropic effects, play a role in the hemostatic system, influence catabolism in muscle and adipose tissue and play a role in cholesterol synthesis and degradation (1, 2). The most common thyroid disorder diagnosed in dogs is hypothyroidism, a clinical condition of low circulating levels of thyroid hormones (2, 3). It is generally thought that decreased thyroid hormone levels play a significant role in the lower metabolic rate in dogs and has been rarely examined in population studies comparing metabolizable energy intake to serum thyroid status. Sighthounds, such as young, healthy racing greyhounds and northern arctic breed dogs, have been found to have lower thyroid hormone concentrations, sometimes below standard reference ranges (2, 3). It has been suggested that breed-specific thyroid hormone reference ranges would be helpful for certain dog breeds, including whippets and Alaskan Husky (AH) sled dogs (4). There is considerable debate surrounding the many possible causes of low thyroid hormone concentrations. Potential contributors of low thyroid hormones include exercise, medications, age-related change, environmental temperature, circadian rhythm effect, seasonal differences, macronutrient differences or micronutrients (i.e., iodine) in the diet, and a negative energy balance associated with working conditions (2, 3, 5–9). Euthyroid sick syndrome can also confound accurate thyroid function assessments (3).

Evanson and colleagues documented exercise significantly decreased total T4 (TT4) and free T4 (fT4) during peak training compared to the off-season (7). Other work by Panciera and colleagues found reduced thyroid hormone levels (T3 and total T4) after an approximately 1,000-mile race (8). Two studies also found an overall decrease in sled dogs' thyroid hormone concentrations during the off-season and when not racing (1, 8). Thus, suggesting that exercise alone was not responsible for the lower thyroid concentrations and that kennel management, diet, and environment may also play a role in serum thyroid concentrations in endurance racing AH sled dogs.

A year-long study following a population of outdoor kenneled Beagles found that total T4 and free T4 increased in November, possibly due to the needed increased basal metabolic rate during cooler temperatures (6). However, Alaskan Husky sled dogs live outside year-round and adapt to the cooler temperatures of their environment. Aging may also influence circulating thyroid hormone levels, with a mild decrease of T4 observed in older pet dogs (3). However, older healthy non-hypothyroid pet dogs' T4 values will usually fall within the low end of the standard reference range. A study did find that Salukis and Sloughis T4 levels decline at the same rate as non-sight hound breed dogs (2). Aging and inherent breed differences can often result in incorrectly diagnosed hypothyroidism leading to inappropriate thyroid supplementation (10, 11). However, no studies to date have examined thyroid hormone levels in aged Greyhound or Alaskan Husky sled dogs. Currently, no studies have examined metabolizable energy intake and thyroid hormone concentrations of older, inactive kenneled sled dogs in ideal body condition in a thermoneutral environment.

We hypothesized that retired sled dogs at ideal body condition (5/9), regardless of sex, would have predominantly normal serum thyroid parameters and that serum thyroid status would correlate with metabolizable energy consumption based on metabolic body weight and there may be significant correlation with TSH and thyroid hormone status. We also investigated whether age, sex (female spayed, male intact or castrated male), or breed (Alaskan Husky vs. Alaskan Husky + Hound cross) influenced thyroid parameters.

## Materials and Methods

Ninety-Six retired sled dogs from across the country currently being monitored as part of a long-term aging study were included. All dogs were obtained as retirees from 14 kennels within the United States between the ages of 8 and 11 years of age. All Alaskan huskies were from mixed lineages of purpose bred racing sled dogs. The dogs acclimated to the kenneled environment and were housed in an indoor thermoneutral kennel facility (76°F) for at least 1 year prior to our study and were deemed healthy on physical exams at the onset of this study. We examined current medical records to ensure none of the dogs were receiving any medications that would affect thyroid hormone levels and had no prior diagnosis of hypothyroidism. Dogs were not on any concurrent medications or medications within 1 month prior to the blood draws including antibiotics, immunosuppressants, or non-steroidal anti-inflammatory medications. Of the 96 dogs assessed 86 met the inclusion criteria for the study. All of the dogs were on the same diet (Annamaet Extra dry food or ProPlan Chicken and Rice Savory formula canned food). One dog was on a hydrolyzed kibble (Purina HA) due to presumptive IBD and was well controlled on this diet alone and no other concurrent medications. Dogs were fed to achieve ideal body condition (BCS 5/9) for 3 months (12). Once they reached ideal body condition, they were observed for maintenance of body weight and metabolizable energy fed for an additional 3 months before drawing 12-h fasting blood samples between 10 am and 2 pm on two separate mornings to avoid diurnal fluctuations. The New York State Diagnostic Laboratory measured serum concentrations of thyroid-stimulating hormone (TSH), total thyroxine (TT4), free thyroxine (fT4—by equilibrium dialysis), 3,3',5—triiodothyronine hormone (T3), and thyroglobulin antibody. Body condition scores, metabolizable energy fed based on calculation from manufacturer label, breed type (AH vs. AH-Hound mix), age, and sex were also recorded.

TT4, fT4, T3, and TSH were compared to standard established reference ranges from the Cornell Endocrinology Laboratory. Body condition score and weight were averaged at the end of the three-month observation period. Metabolic body weight was calculated based on the equation BW(kg)^0.75^. Metabolic energy was assessed based on the food (Annamaet Senior Dog Food, Sellersville, PA, Proplan Performance Savory Chicken Dog Food, Nestle-Purina; St. Louis, MO, or both) that was fed to each dog based on the manufacturers' assessments from modified Atwater equations as kilocalories per cup or per can fed to each dog as one daily meal. The diet fed is an American Association of Feed Control Officials approved diet (adequate in all nutrients including iodine) which is sold nationally. Metabolizable energy intake was then divided by metabolic body weight to provide kilocalories consumed per kilogram of metabolic body mass. Pearson's correlations were performed on kilocalories fed on a metabolic body weight basis to assess the relationship with thyroid hormone concentrations and for assessment of TSH correlation to serum thyroid hormone indices. We calculated R values, assessed as R <0.3 being weak correlations, *R* > 0.3–0.5 modest correlations, *R* > 0.5−0.7 moderate correlations, and >0.7 strong correlations. Sex, breed type, and age were assessed for normality of hormone concentrations utilizing a Shapiro Wilks test. Numerical data are presented as mean +/- standard deviation. A *T*-test for two way comparisons or ANOVA for greater than two group comparisons for TT4, fT4, and T3, concentrations across sex (MN, MI, FS) breed type (Alaskan Husky vs. Alaskan husky + Hound mix) and age (<10, 11, 12, and 13). A *P*-value of <0.05 established significance.

## Results

**Figure 1:**
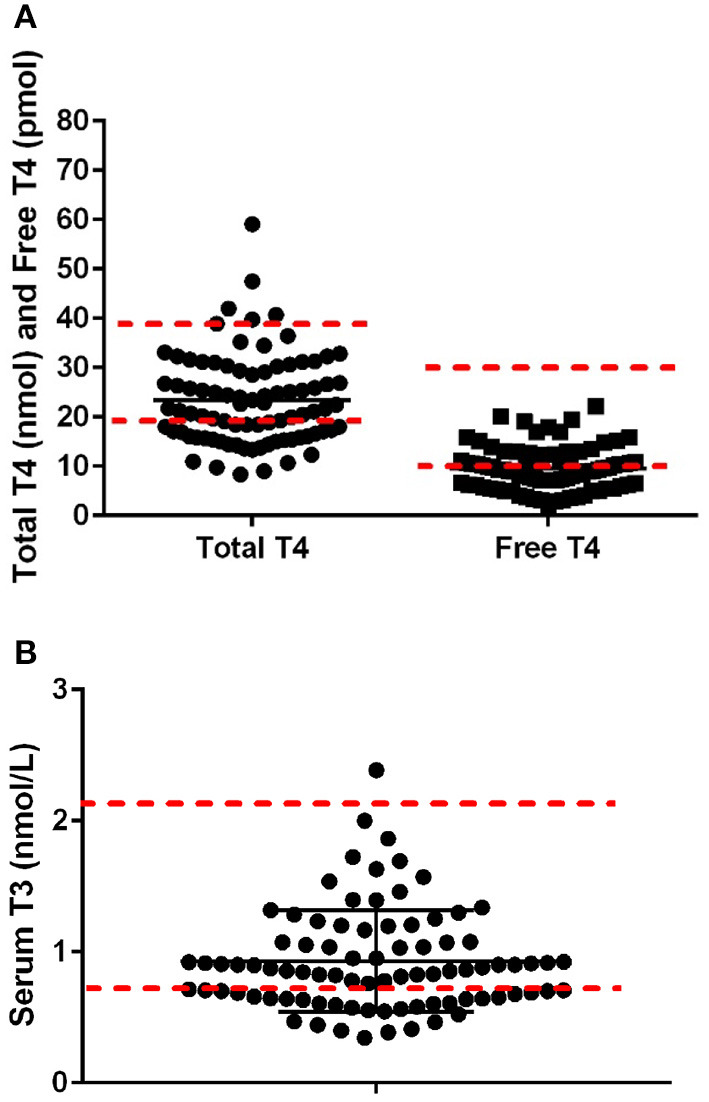
**(A)** Serum concentrations of total thyroid hormone concentration (range 18–38 nmol/L) and free thyroid hormone (range 8–28 nmol/L; n-−86) in Alaskan Huskies. **(B)** Serum concentrations of T3 hormone concentration (range 0.8–2.1 nmol/L; n-−86) in aging Alaskan Huskies. Red dashed lines are the lower higher reference range based on Cornell University Diagnostic Laboratory Endocrinology Lab.

**Figure 2:**
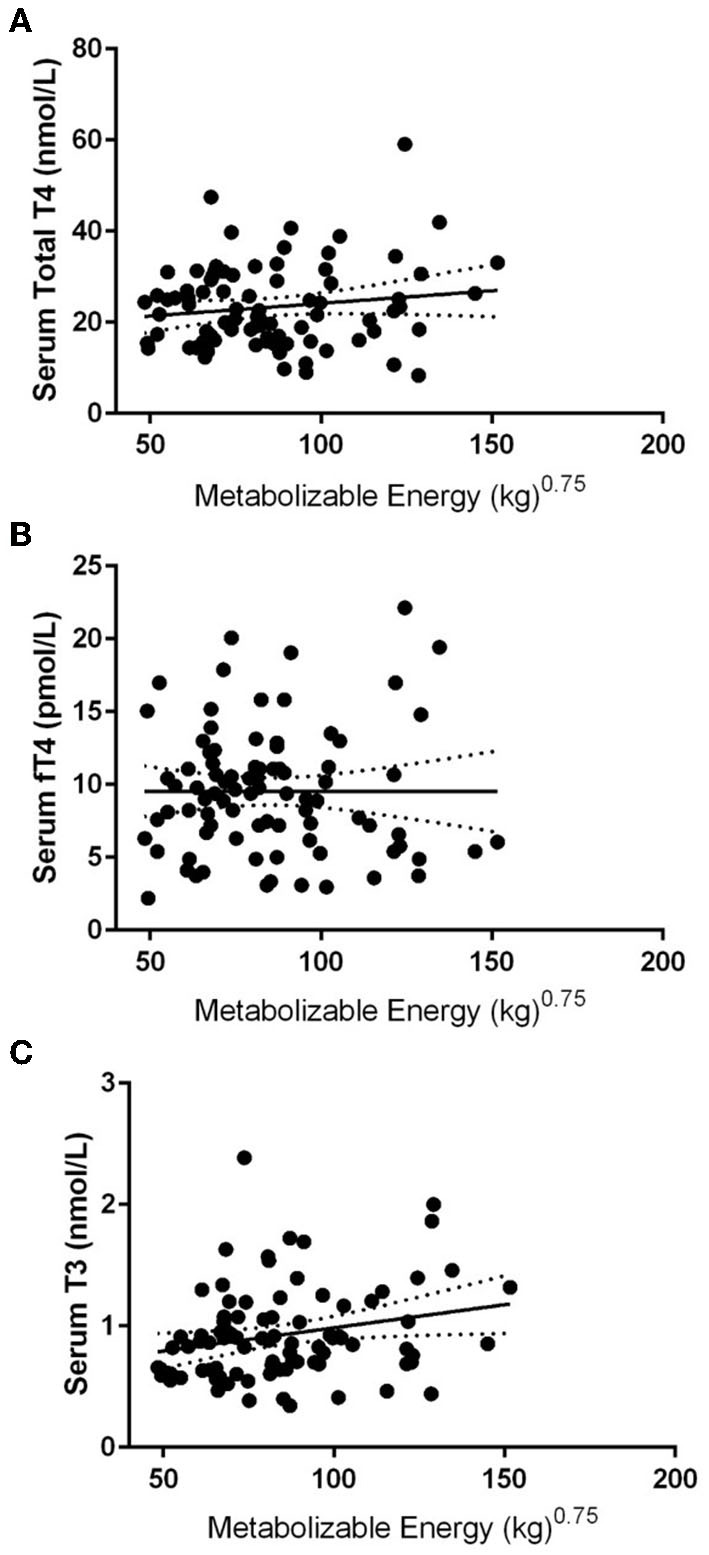
Pearson linear regression statistics comparing various thyroid hormone concentrations vs. metabolizable energy intake in 86 Alaskan Sled Dogs. **(A)** Linear regression with 95% confidence intervals of TT4 to metabolizable energy showing weak insignificant correlation (*R* = 0.14). **(B)** Linear regression analysis with 95% confidence intervals of fT4 to metabolizable energy showing weak insignificant correlation (*R* = 0.01). **(C)** Linear regression analysis with 95% confidence intervals of T3 to metabolizable energy showing weak significant correlation (*R* = 0.23: *p* = 0.03).

After exclusion based on medication status and negative for thyroglobulin antibody and TSH results within normal ranges, 86 dogs were included in the data analysis. The mean body condition score was 5.1 ± 0.4, and the mean body weight was 24.5 ± 4.2 kg at the time of blood draws. Mean serum TT4, free T4, and T3 were 23.4 ± 9.1 nmol/L, 9.53 ± 4.3 pmol/L, and 0.93 ± 0.39 nmol/L, respectively, with 38% of the dogs being lower than the reference range for total TT4, 45% for fT4, and 37% for T3 (Figures 1A,B). Pearson's correlates were calculated based on kilocalories consumed on a metabolic body weight basis for TT4 (*R* = 0.14), fT4 (*R* = 0.01), and T3 (*R* = 0.23), showing poor correlation; with only T3 showing significance (*p* = 0.03: Figures 2A–C). Additionally, correlation assessment of TSH status on serum fT4 (*R* = 0.14) TT4 (*R* = −0.28) and T3 (*R* < 0.01) were considered weak and not statistically significant.

Sixty-three AH and 23 AH-hound mixed breed dogs were assessed for serum thyroid parameters. Normality testing using Shapiro Wilks testing revealed normality of the data set when assessing sex, breed, and age across groupings leading to parametric statistical testing (ANOVA or unequal variance *T*-Tests). The TT4, fT4 and T3 between AH (22.6 ± 8.7 nmol/L; 9.12 ± 4.25 pmol/L; 0.91 + 0.37 nmol/L) vs. AH-Hound mixes (25.37 ± 10.0 nmol/L; 10.66 ± 4.47 pmol/L; 0.97 ± 0.43 nmol/L) were not significantly different. Across the population there were twenty-four dogs that were 10 years or younger, thirty-eight 11 year old dogs, sixteen 12 year old dogs and nine 13 year old dogs. The TT4, fT4, and T3 were not significantly different across age stratified at ≤10 (22.0 ± 7.1 nmol/L; 9.8 ± 3.8 pmol/L; 1.0 ± 0.31 nmol/L), 11 (24.1 ± 10.7 nmol/L; 9.51 ± 4.72 pmol/L; 0.86 ± 0.38 nmol/L), 12 (24.9 ± 9.3 nmol/L; 10.66 ± 4.67 pmol/L; 0.96 ± 0.52 nmol/L) or 13 (20.0 ± 5.7 nmol/L; 6.72 ± 3.01 pmol/L; 0.86 ± 0.40 nmol/L) years of age. As all females in the colony were spayed ~1 year or more prior to assessment the population consisted of 42 FS, 11 MC, and 34 MI dogs. When assessing sex, no significant differences were observed in TT4, fT4, or T3 across FS (23.9 ± 8.4 nmol/L; 9.82 ± 4.11 pmol/L; 0.86 ± 0.38 nmol/L), MC (21.6 ± 6.4 nmol/L; 7.87 ± 3.2 pmol/L; 0.80 ± 0.37 nmol/L) or MI (23.2 ± 10.7 nmol/L; 9.74 ± 4.88 pmol/L; 1.05 ± 0.37 nmol/L).

## Discussion

Our study sought to examine the relationship between thyroid hormones and dietary energy intake, age, breed, and sex in a cohort of AH dogs. Interestingly, we did not find any robust correlation between thyroid parameters and metabolic energy requirements in our cohort. Notably, thyroid hormone concentrations were measured below the reference interval in many dogs in our sled dog population without increased TSH to implicate hypothyroidism. TSH elevations are routinely used to help rule in clinically significant thyroid disease as a dysfunctional negative feedback, however we found no correlation to serum thyroid hormone status which is not surprising as TSH is 100% sensitive, yet only 60% specific for diagnosis of thyroid disease making our findings realistic (13).

Eales' 1988 literature review concluded that “mammalian thyroidal response to food ingestion is complex and involves many interrelated levels of thyroid function (14).” The exact relationship between food ingestion and thyroid hormone levels is not entirely understood. Eales found that thyroid T4 and T3 levels decreased in fasted dogs to a similar degree as fasted humans. He postulated that the production of T4 and T3 could be related to energy intake, especially carbohydrates. Daminet et al. examined thyroid hormones in obese dogs, lean dogs, and obese dogs while on a weight loss diet and found that total T3 and T4 were higher in obese dogs than lean dogs while remaining within the normal reference range (15). Interestingly, the dogs with higher T3 levels required more time to achieve ideal weight regardless of starting body condition score (15). Dogs consuming a weight loss diet had lower total T3, TT4, and TSH concentrations; however, significance was only observed in total T3 and TSH. Our results showed a very weak correlation between metabolizable energy and T3 thyroid hormone, suggesting a potential association similar to the findings of Daminet and colleagues'. Daminet et al. postulated that the lower T3 observed was due to undernutrition. A low total T3 is commonly seen in euthyroid sick syndrome dogs; however, neither undernutrition nor euthyroid sick syndrome were factors in our study (15).

Other possible hormones that could be affecting T3 include testosterone and cortisol. Prior research found that thyroid hormones have a role in regulating semen quality by altering testosterone levels in men and young boys (16). In one study, healthy azoospermic Labrador retrievers had higher thyroid levels and lower testosterone levels (17). These thyroid hormones were high normal, or slightly above the normal reference range. Our study evaluated a mixed population of male and female sled dogs, some of which were neutered males, with lower thyroid hormones than the reference range. None of the intact males had higher thyroid hormone concentrations than neutered males or spayed females. This discordance may be due to our population's difference in breed and age compared to the Labrador study or the late age when most dogs in our study were sterilized. Unfortunately, little is known about the effects of estrogen on thyroid hormone status and our cohort was spayed not allowing for any associations to be derived revealing a limitation of the study which cannot be addressed. Additionally, our population of neutered males vs. intact males was skewed toward far more intact males which makes our results somewhat tenuous and further study in intact males and females vs. non-sexually intact dogs is worth further study.

Glucocorticoids suppress the hypothalamus-pituitary-thyroid axis reducing thyroid hormone levels. High endogenous cortisol was linked to a significant decrease in TT4, and fT4 to a lesser extent, while an anti-inflammatory dose of prednisone caused a decrease in T3 (18). The present study excluded all dogs with any indication of hyperadrenocorticism or were receiving any exogenous prednisone. Therefore, our results of low thyroid hormones are not iatrogenic in nature, with just over 1/3^rd^ of our population having low TT4 levels and nearly half displaying low free T4 concentrations. Other medications such as phenobarbital, potassium bromide, non-steroidal anti-inflammatory drugs, and sulfonamides can cause lower thyroid hormones (19). The dogs evaluated in our study were not receiving any such medications, suggesting a global breed effect. We postulate that much of the prior literature in sled dogs documenting lower than normal serum thyroid condition could have been due to the exercise and intermittent negative energy balance. Our colony was kept within ideal body condition 5/9 and were not exercised and still showed even greater percentages of dogs with low serum thyroid status strongly implicates a breed or age effect.

Most other studies evaluating thyroid hormone levels in Alaskan Huskies and other sighthounds are conducted in younger dogs that are still active in sport or as companions. This study is the first to examine geriatric sled dogs, which may influence our reported range. The Scott-Moncrieff review stated that a progressive decline in T4 is seen in older healthy dogs as an age-related change (2). This was examined in Beagles and Labradors over the age of 6 years and found to have decreased fT4, TT4, and T3 in the older group compared to dogs under 6 years old (2). Scott-Moncrieff cited several possible reasons for this decline, including altered responsiveness of the thyroid gland, decreased biologic activity of TSH with age, and subclinical thyroid pathology (2). In the German Shepherd dog, a study of 57 dogs stratified into six and under and over 6 years of age found that aging dogs tended to have higher TSH and lower fT4 suggesting that aging influences the hypothalamic-pituitary-thyroid axis (10). While a decrease in thyroid hormones occurred in older German Shepard dogs, the values still fell in the lower portion of the normal reference range. Our present study in older sled dogs consistently showed thyroid levels well below the reference range. Compared to the studies mentioned above, age was not associated with significant differences in thyroid hormone concentrations in our cohort. This may be due to our study population's limited age range of 8–13 years. Further examination is needed on a larger sled dog population stratified by age to determine if the thyroid values reported here accurately represent this breed and age group.

There is speculation that colder environmental temperatures and exercise could influence the carrier protein binding of T3 and T4 yielding lower values (8). It has also been proposed that the high-fat diets of competing endurance Alaskan Husky sled dogs could also contribute to lower protein binding of T3 and T4 due to increased amounts of free fatty acids displacing them (8). Panciera et al. also mentioned that mushers competing in the same environment as the Alaskan Husky sled dogs showed no difference in the values of T3, T4, fT3, or fT4 after a long-distance race giving further support that temperature alone is not responsible for globally lower thyroid hormone concentrations (8). Oohashi et al. studied seasonal influences on thyroid hormone levels in healthy outdoor dogs and found an increased fT4 and TT4 levels in November (6). They concluded that this increase was to increase the basal metabolic rate in the face of colder temperatures to maintain normothermia. Sled dogs typically live outside all year long and are very accustomed to the temperatures. The geriatric sled dogs in this study live indoors in an environment closer to what pet companion dogs live in, therefore eliminating temperature differences as an influence in our study and further suggesting that environment is not a major contributor to chronically low thyroid status in our study.

A complicating factor that we considered is that the AH breed has diverged in the past 20 years with the breeding of hounds, primarily the German Shorthaired and English Pointer, into the AH. Based on pedigree assessment, a portion of our population has at least 1/16th hound up to 3/8th hound as part of their pedigree. Therefore, we designated these dogs as AH-Hound mixes to compare to dogs from pure AH pedigrees. A recent study examining thyroid parameters found that TT4 and fT4 were significantly lower in the English Setter, Golden Retriever, and Collie than Alaskan Malamute and the Siberian Husky breeds with intermediate concentrations. Both groups differed from the Keeshond and Samoyed with the highest breed concentrations (11). Our lack of difference suggests that the penetrance of the pointer into the AH may not be sufficient to alter these concentrations when grouped, or that the Pointer breeds may also be considered on the lower end regarding serum thyroid status, which would require further investigation comparing these breeds to the traditional AH.

In conclusion, no correlation was found between the metabolizable energy and thyroid hormone concentrations. Furthermore, when compared by age, breed, or sex, there were no differences in thyroid concentrations in geriatric AH sled dogs. Environmental and metabolic factors were eliminated since the study dogs were housed in a normothermic environment and screened for abnormalities on physical exam and blood work. These results lead us to believe breed-specific thyroid hormone ranges are necessary to avoid misdiagnosis of hypothyroidism in sled dogs. Although supplementation due to this mild to moderate diminished thyroid hormone status is unlikely to be detrimental, it is more likely that clinical signs and corresponding bloodwork should be evaluated thoroughly in this breed to better understand whether supplementation is necessary considering the cost for monitoring supplementation and medicating daily.

## Data Availability Statement

The raw data supporting the conclusions of this article will be made available by the authors, without undue reservation.

## Ethics Statement

The animal study was reviewed and approved by Cornell University Institutional Animal Care and Use Committee.

## Author Contributions

The research idea was the genesis of all authors. HH, JL, JW, and ML performed data analysis and acquisition. All authors contributed to the drafting and revision of this manuscript. All authors contributed to the article and approved the submitted version.

## Funding

This work was funded by the non-for-profit Vaika Foundation.

## Conflict of Interest

AG, KA, and DF were employed by Vaika Foundation. The remaining authors declare that the research was conducted in the absence of any commercial or financial relationships that could be construed as a potential conflict of interest.

## Publisher's Note

All claims expressed in this article are solely those of the authors and do not necessarily represent those of their affiliated organizations, or those of the publisher, the editors and the reviewers. Any product that may be evaluated in this article, or claim that may be made by its manufacturer, is not guaranteed or endorsed by the publisher.
